# Pharmacotherapy for primary biliary cholangitis: an assessment of medication candidacy and rates of treatment

**DOI:** 10.1186/s12876-023-03108-4

**Published:** 2024-01-04

**Authors:** Nicholas MacDonald, Rebecca Loh, Jonathan M. Fenkel, David A. Sass, Dina Halegoua-DeMarzio

**Affiliations:** 1 Department of Medicine, Thomas Jefferson University Hospital, 33 S 9 TH St, Suite 220, 19107 Philadelphia, PA USA; 2 Department of Medicine, Division of Gastroenterology and Hepatology, Thomas Jefferson University Hospital, 132 South 10th Street, Suite 480, 19107 Philadelphia, PA USA

**Keywords:** Primary biliary cholangitis, Cirrhosis, Ursodeoxycholic acid, Obeticholic acid, Fibrate

## Abstract

**Background:**

Ursodeoxycholic acid is the preferred first-line therapy for primary biliary cholangitis. Alternative therapies, such as obeticholic acid, are recommended for patients who cannot tolerate ursodeoxycholic acid or who have an inadequate response to ursodeoxycholic acid monotherapy. Prior investigations have suggested that as many as 30% of patients with primary biliary cholangitis may have never received treatment with ursodeoxycholic acid. No prior investigations have examined usage rates of obeticholic acid in the treatment of primary biliary cholangitis.

**Methods:**

All patients with an ICD-10 diagnosis of primary biliary cholangitis who had any records within the health system were included. A review of medical records was performed to confirm the diagnosis of primary biliary cholangitis and determine which medications had been prescribed for treatment, as well as candidacy for second-line therapies.

**Results:**

A total of 495 patients met inclusion criteria. Notably, 95% of patients were taking ursodeoxycholic acid for treatment of their primary biliary cholangitis, with 67% of patients having disease that was well-controlled on ursodeoxycholic acid monotherapy. In total, 8% of patients were taking obeticholic acid (either as combination or monotherapy). Only 3% would benefit from the addition of a second line therapy but had not yet been offered medication. Only 3% of patients were not on any medication for management of their primary biliary cholangitis.

**Conclusions:**

Ursodeoxycholic acid is a readily available and generally well-tolerated medication that should be offered to all patients with primary biliary cholangitis as first-line therapy. While prior investigations have suggested that up to 30% of patients with primary biliary cholangitis may never have received treatment for the disorder, the present study suggests that patients are generally being managed according to guidelines. Moreover, a significant proportion of patients with primary biliary cholangitis will qualify for second line therapies and prescribers should be aware of the indications to use these medications.

## Background

Primary Biliary Cholangitis (PBC) is a chronic cholestatic liver disease involving the autoimmune-mediated destruction of biliary epithelial cells. PBC is a progressive disorder, and may ultimately result in hepatic fibrosis, cirrhosis, and decompensated liver disease [1]. Patients are frequently asymptomatic from the disorder for a period of years or even decades. If symptoms do eventually develop, they are most commonly pruritus and fatigue and less commonly jaundice or abdominal pain [2]. Ursodeoxycholic acid (UDCA) is the preferred, first-line pharmacologic agent for treatment of PBC and is indicated in all patients with the disorder. UDCA is administered at a dose of 13–15 mg/kg/day, typically divided into 2–4 doses per day. The medication is generally well tolerated and, when used for PBC, the only true contraindication is hypersensitivity. In the absence of UDCA pharmacotherapy, the median survival for patients with PBC ranges from 5 to 8 years from the onset of symptoms [3–6]. Treatment with UDCA has been consistently shown to improve biochemical indices, delay histologic progression, delay development of esophageal varices, and improve transplant-free survival in patients with PBC [7–10].

However, despite the efficacy of UDCA in the treatment of PBC, approximately 40% of people will respond incompletely to the drug as monotherapy [11]. Recent investigations have demonstrated the efficacy of alternative treatments for PBC. Obeticholic Acid (OCA) received FDA approval in 2016 after the POISE trial showed that 46–47% of patients receiving the medication were able to achieve reduction in alkaline phosphatase to less than 1.67 times the upper limit of normal and normalization of bilirubin compared to 10% of patients in the placebo group [12]. Pruritus is a common adverse effect that limits the use of OCA in some patients, but the medication is otherwise generally well-tolerated [13]. Fibrates also have an off-label indication for treatment of PBC given their anticholestatic properties [14]. At present, alternative therapies are only recommended for patients who cannot tolerate UDCA or who demonstrate an inadequate response to UDCA monotherapy. Furthermore, based on an FDA restriction published in 2021, obeticholic acid (OCA) is contraindicated in any patient with advanced cirrhosis, defined as Child-Pugh class B or C cirrhosis, known portal hypertension, and/or any history of liver decompensation [15]. In spite of UDCA’s proven efficacy in the treatment of PBC, recent studies have demonstrated that as many as 30% of patients with PBC may never have received appropriate treatment with UDCA and further suggest that these differences vary on the basis of demographic factors like age, sex, and race [16–18]. To the authors’ knowledge, however, no study has yet quantified usage rates of second line therapies for PBC. The aim of this study is to characterize usage rates of UDCA and second line therapies among patients with PBC.

## Methods

This was an observational, cross-sectional study performed at a large urban health system with an academic liver transplant program. Patients were identified in the electronic medical record according to the ICD-10 code for PBC (ICD-10-CM: K74.3). All patients with a diagnosis of PBC who had any records within the health system between 2017 and 2022 were eligible for inclusion. A review of medical records was then performed to confirm the diagnosis of PBC. PBC was defined according to the American Association for the Study of Liver Diseases (AASLD) practice guidelines as meeting two of the following three criteria: biochemical evidence of cholestasis based on alkaline phosphatase elevation; presence of Anti-Mitochondrial Antibodies (AMA) or other PBC-specific autoantibodies (including sp100 or gp210, if AMA is negative); histologic evidence of nonsuppurative destructive cholangitis and destruction of interlobular bile ducts. Patients were excluded if they were deceased at the time of chart review or if they had ever received a liver transplant. Review of medical records also included a determination of which medications had been prescribed for the treatment of each patient’s PBC (if any), as well as candidacy for second-line therapies. Patients were considered a candidate for OCA if they were unable to tolerate UDCA or their alkaline phosphatase did not decrease to less than 1.67 times the upper limit of normal after one year of therapy with UDCA at appropriate weight-based dosing, and they did not have advanced liver disease that would preclude the use of OCA [1].

## Results

**Fig. 1:**
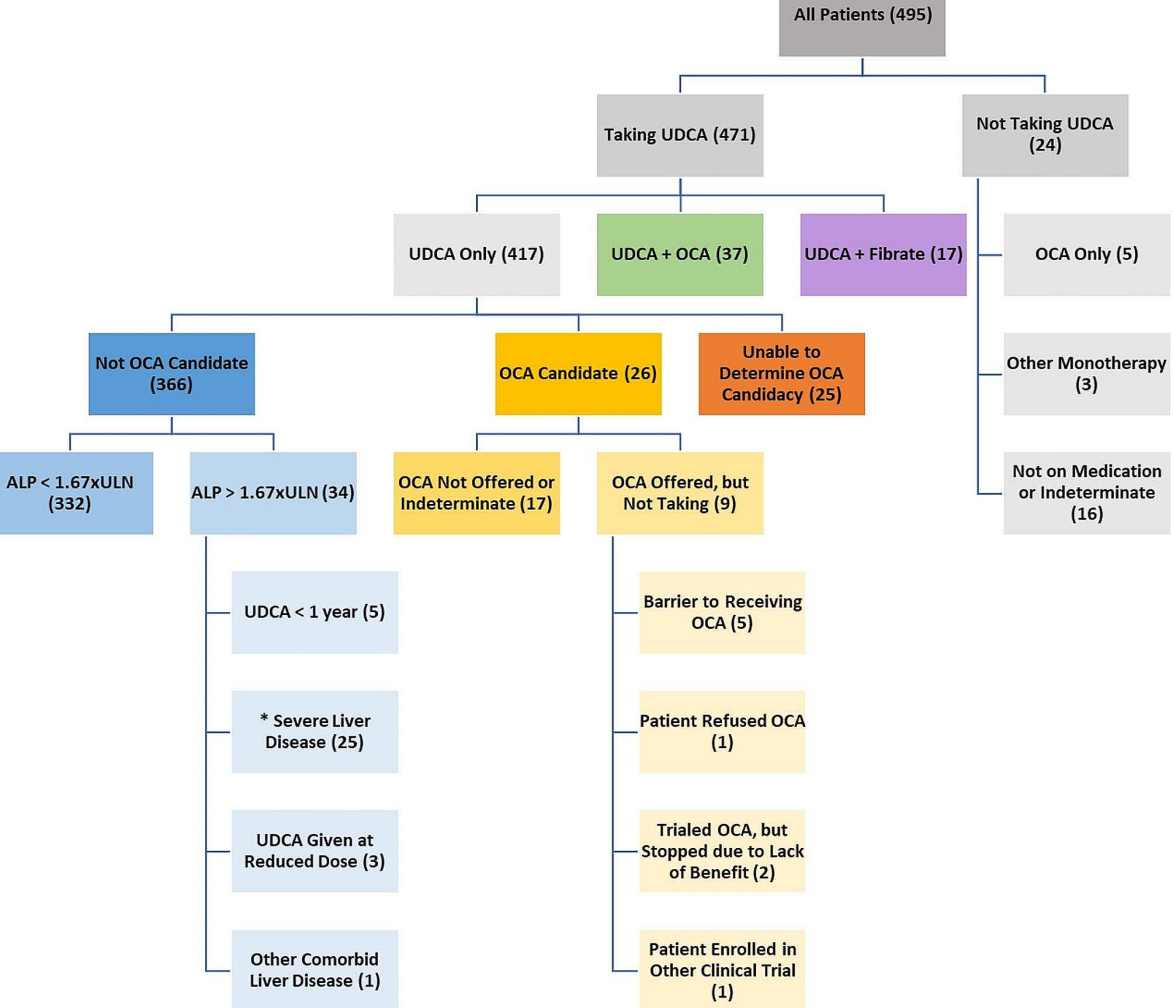
Medication candidacy and usage among patients diagnosed with PBC. UDCA– Ursodeoxycholic Acid; OCA– Obeticholic Acid; ALP– Alkaline Phosphatase; ULN– Upper Limit of Normal. Shown are all patients with a diagnosis of PBC who have records within the health system. Patients were stratified on the basis of medication usage and candidacy for OCA. The number of patients in each category is displayed in parentheses. OCA candidacy is considered to be one or more of the following: failure of ALP to decrease to within 1.67 times the upper limit of normal after one year of therapy with UDCA at appropriate weight-based dosing; inability to tolerate UDCA at appropriate weight-based dosing. *Severe liver disease is defined as one or more of the following: Child-Pugh B/C categorization; presence of portal hypertension; history of liver decompensation. Severe liver disease is a contraindication to the use of OCA.

At the conclusion of chart review, 495 patients met inclusion criteria for this study. Of these 495 patients, 91% self-identified as female, 7% as male, and 2% were unknown. Likewise, 78% self-identified as white/Caucasian, 7% as black/African American, 3% as Asian/American Indian/Pacific Islander, and 11% were unknown. In total, 6% self-identified as Hispanic (Table 1). Within the study population, 95% of all patients had been prescribed UDCA for treatment of their PBC. It was determined that 67% of all patients studied had PBC that was well controlled on UDCA monotherapy, as defined by an alkaline phosphatase level less than 1.67 times the upper limit of normal after 1 year of UDCA at appropriate weight-based dosing. While 14% of all patients were determined to be an appropriate candidate for OCA, only 8% of all patients were taking the medication − 7% were taking OCA in combination with UDCA and 1% were using OCA as monotherapy due to intolerance of UDCA. Likewise, 3% of all patients were on combination therapy with UDCA and a fibrate, though in some cases it was not evident from chart review whether a fibrate had been prescribed as treatment for PBC or as treatment for some other disease process (e.g. dyslipidemia). In only 3% of all cases were patients either not on therapy (typically related to issues with medication adherence or follow-up) or there was inadequate documentation to determine whether any treatment had been discussed or prescribed. Notably, only 3% of all patients studied (17 individuals) had a persistently elevated alkaline phosphatase level despite appropriate treatment with UDCA and would benefit from the addition of a second line drug but had not been offered the medication by a prescriber (Fig. 1). In total, 18% of all patients studied had at least one contraindication to OCA (Child-Pugh class B or C cirrhosis, known portal hypertension, and/or any history of liver decompensation).

**Table 1:** Self-reported demographic information of study participants

Demographics	Number of Individuals (n = 495)
Female	450 (91%)
Male	35 (7%)
Unknown	10 (2%)
White/Caucasian	388 (78%)
Black/African American	36 (7%)
Asian/American Indian/Pacific Islander	16 (3%)
Unknown	55 (11%)
Hispanic	30 (6%)

## Discussion

The purpose of this study was to quantify candidacy and usage rates of pharmacotherapy in patients with a diagnosis of primary biliary cholangitis. A total of 495 patients were studied using medical records from a large urban health system with an academic liver transplant program. A female-to-male ratio of 13:1 for the prevalence of the disorder was identified. This was slightly higher than the oft-reported 9:1 female-to-male ratio of PBC prevalence that has been demonstrated in prior demographic analyses of the disorder [1]. Our study population was predominately female and white/Caucasian, though a diagnosis of PBC was present in all sexes and ethnicities. As much of the data regarding PBC has historically been collected from predominately Caucasian populations, limited data exist regarding disease prevalence in other groups in the United States. However, a recent study of 4241 PBC patients nationwide found that 64% identified as white, 8% African American, 7% Asian American/American Indian/Pacific Islander, and 21% were unknown. Of these, 21% self-identified as Hispanic [16].

PBC is a chronic disorder that can progress to cirrhosis and decompensated liver disease. In the absence of appropriate treatment, the median survival is only 5 to 8 years from the onset of symptoms. UDCA is a readily available and generally well-tolerated medication that has been consistently shown to improve mortality in PBC and should be offered to all patients as first-line therapy. Despite prior investigations identifying that as many as 30% of patients with PBC may have never received treatment with UDCA, the present study found that 95% of patients were taking UDCA as part of their therapy, and only 3% of patients were not on any medication for the disorder [16–18]. In the population studied here, two-thirds of all patients had disease that was well controlled using UDCA as a single agent. In total, 14% of patients studied were appropriate candidates for OCA, the preferred second-line agent for treatment of PBC. Prompt recognition of these patients is vital, as OCA is contraindicated in any patient whose liver disease has progressed to the point of causing Child-Pugh Class B or C cirrhosis, portal hypertension (which can occur in PBC even prior to the development of cirrhosis), [19] or any form of hepatic decompensation. Notably, 18% of all patients studied had liver disease that met one or more of these criteria and would no longer be considered candidates for OCA, even if they exhibited poor disease control with UDCA monotherapy or intolerance of UDCA.

A significant limitation of this study may be that it was conducted within a single urban health system, which may be further biased by the presence of an academic liver transplant program. However, consultation with a hepatologist was not a requirement for inclusion in this study. Moreover, all patients with any records within the health system were considered eligible for inclusion in this study, regardless of where they may receive the majority of their healthcare. Access to health information exchanges allowed the authors to capture a broader patient population, including those whose PBC was being managed by clinicians at other health systems. As has been identified in prior analyses, the majority of patients studied here identified as both female and white/Caucasian. Unfortunately, the sample size was insufficient to draw meaningful conclusions regarding discrepancies in medication candidacy or usage between demographic groups. This may represent a future area of study.

## Conclusions

UDCA is a readily available and generally well-tolerated medication that should be offered to all patients with PBC as first-line therapy. While prior investigations have suggested that up to 30% of patients with PBC may never have received treatment for the disorder, the present study suggests that patients are generally being managed according to guidelines. Moreover, a significant proportion of patients with PBC will qualify for second line therapies and prescribers should be aware of the indications to use these medications.

## Data Availability

The datasets generated and analyzed in the present study are included in aggregate form within the manuscript. Individual data are not publicly available as they represent protected health information.

## Abbreviations

AASLD: American Association for the Study of Liver Diseases
AMA: Anti-Mitochondrial Antibody
FDA: Food and Drug Administration
ICD: International Classification of Diseases
OCA: Obeticholic Acid
PBC: Primary Biliary Cholangitis
UDCA: Ursodeoxycholic Acid
